# The complete chloroplast genome of the common self-heal, *Prunella vulgaris* (Lamiaceae)

**DOI:** 10.1080/23802359.2018.1424587

**Published:** 2018-01-11

**Authors:** Yu-Wei Han, Ting-Yun Zheng

**Affiliations:** State Key Laboratory of Quality Research in Chinese Medicine, Institute of Chinese Medical Sciences, University of Macau, Macau, China

**Keywords:** *Prunella vulgaris*, medicinal herb, chloroplast genome, phylogenomics

## Abstract

*Prunella vulgaris* L. is an important medicinal herb widely used in China and western countries. Its natural distribution occurs in various habitats throughout northern hemisphere. In present study, we assembled and characterized its whole chloroplast (cp) genome based on Illumina pair-end sequencing data. The complete chloroplast genome size is 156,132 bp. It contained 134 genes, including 89 protein-coding genes, 37 tRNA genes, and 8 rRNA genes. 8 gene species had two copies. The overall GC content of this genome was 37.9%. A further phylogenomic analysis of Lamiaceae, including 29 taxa, was conducted for the placement of *P. vulgaris*.

*Prunella vulgaris* L. (Lamiaceae), is a perennial herb known as ‘common self-heal’ found in temperate to subtropical climates throughout Europe, Asia, and North America. It is a long used herbal medicine in both Europe and China for treatment of fevers, sore mouth, and wound healing (Kim et al. 2014; Lou et al. 2014; Qu et al. 2017). In the present study, we assembled and characterized the complete plastome of *P. vulgaris.* It will provide potential genetic resources, such as SNPs, DNA barcodes and cpSSR markers, for further population genetics and germplasm diversity conservation studies.

Fresh leaves of *P. vulgaris* were collected from Zhejiang province, China. A voucher specimen (accession number HYW170001) has been deposited at the University of Macau. The total DNA of *P. vulgaris* was extracted using CTAB. A short-insert (500 bp) paired-end library was prepared and sequenced using by HiSeq^TM^ 2500 (Illumina, San Diego, CA, USA). Approximately 16 million high quality clean reads were generated. Then the cp genome was aligned, assembled, and annotated by CLC Genomics Workbench v7.5 software (CLC Bio, Aarhus, Denmark), GeSeq (Tillich et al. 2017), tRNAscan-SE v1.3.1 (Schattner et al. 2005). The rRNAs and protein-coding genes (PCGs) were further validated by comparing with a custom BLAST database of Lamiaceae plastomes annotations.

The complete cp genome of *P. vulgaris* (GenBank Accession MG589640) was 151,342 bp in size, containing a large single copy region (LSC) of 82,689 bp and a small single copy region (SSC) of 17,428 bp, which were separated by a pair of inverted repeats (IRs) of 25,613 bp. The cp genome contained 134 genes, including 89 protein-coding genes (PCGs), 37 tRNA genes, and 8 rRNA genes. Most of the genes occurred as single-copy, while 20 genes had two copies, which included 9 PCG genes (*ndhB*, *rpl2*, *rpl23*, *rps7*, *rps12*, *rps19*, *ycf1*, *ycf2*, and *ycf15*), 7 tRNA genes (*trnA-UGC*, *trnI-CAU*, *trnI-GAU*, *trnL-CAA*, *trnN-GUU*, *trnR-ACG*, and *trnV-GAC*), and all 4 rRNA species (*rrn4.5*, *rrn5*, *rrn16* and *rrn23*). Two PCGs (*clpP*, and *ycf3*) contained two introns while another 8 genes (*atpF*, *ndhA*, *ndhB*, *petB*, *petD*, *rpl2*, *rpl16*, *rpoC1* and *rps16*) had one intron each. The overall GC content of the *P. vularis* cp genome was 37.9%, with the corresponding values of LSC, SSC and IR regions being 36.0, 31.8 and 43.1%, respectively.

The phylogenetic analyses were conducted based on 86 protein-coding genes of 29 complete cp genomes in Lamiaceae to uncover the phylogenetic placement of *P. vulgaris*. The maximum likelihood (ML) inference was performed using GTR + Γ model with 1000 bootstrap replicates in RAxML v.8.2.8 (Stamatakis 2014). The result revealed that *P. vulgaris* was clustered with a clade of *Dracocephalum* + * Mentha* with 100% bootstrap values (Figure 1). In conclusion, the complete cpDNA of *P. vulgaris* is decoded for the first time in this study and provides essential and important DNA molecular data for further phylogenetic and evolutionary analysis.

**Figure 1.**
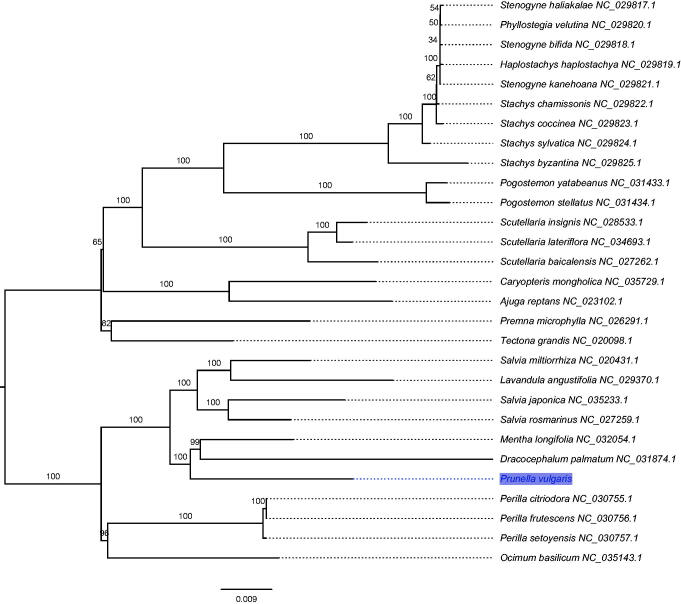
The maximum likelihood (ML) tree inferred from 29 chloroplast genomes. The position of *Prunella vulgaris* is shaded and bootstrapping values are listed for each node.
